# Employment of the Salicylate of Soda for Rheumatism, Gout, Etc., in Scotland, France and Germany

**Published:** 1878-01

**Authors:** W. S. Caldwell

**Affiliations:** M. D., of Warren, Ills.; Paris


### LETTER FROM PARIS.—THE EMPLOYMENT OF THE SALICYLATE OF SODA FOR RHEUMATISM, GOUT, ETC., IN SCOTLAND, FRANCE, AND GERMANY.

There is probably no disease in the whole nosology, that has had so many vaunted remedies brought forward from time to time for its treatment, as acute rheumatism; each in its turn enjoying a brief period of popularity, and sinking into obscurity after being tested and failing to meet the expectation of the profession.

Most of us who have been in practice for a term of years, can remember some case of this disease, in the management of which we have failed to secure the good opinion of our patient.

Among the last of the remedies that have been brought to our notice for the treatment of this most troublesome disease, are salicin and the salicylates.

Since my arrival in Europe, I have taken every opportunity to consult the leading physicians in the different hospitals that I have visited, in order to gain from them their views in regard to the efficacy of these remedies in the treatment of the above disease.

My first observations and inquiries were made at Edinburgh and Leeds, and the facts that I there elicited were published in an article that I contributed to the New York *Medical Record* of Sept 1st, 1877, and from which I shall draw in part in what I shall have to say in this communication.

In Edinburgh I found the remedy in poor repute.

Prof. Sanderson declared to me that, after trying the salicylic acid to his entire satisfaction, he had become convinced that it was worse than useless in the treatment of acute rheumatism, on account of its tendency to derange digestion, and lower the vitality of the patient, without eradicating the disease for which it was given.

After testing alkalies with the same unsatisfactory results, he now treats the disease entirely expectantly, shielding the parts involved from the air by carded wool, over which he applies oiled silk, administering anodynes to relieve pain when urgently demanded.

But, from what I have since learned, I am more than suspicious that his want of success arose from his not using the remedy in an efficient manner. Moreover, the preparation that he employs is not that which is preferable.

It will be seen from the title of this article, that I refer only to salicylate of soda. I do so in order to impress the reader with the idea, that this preparation of the salicylates, and this one *only*, should be used in the treatment of acute rheumatism, for reasons that I shall take occasion to mention.

In Dr. Jacobhouse, physician to the Leeds Infirmary, I found one of the most enthusiastic students I ever met, and would add that he who travels through Europe for the purpose of making hospital and clinical observations, and does not, when in this city, visit the Leeds Infirmary, will have reason to regret it.

Dr. Jacobhouse, when I first made his acquaintance, had just concluded a series of observations on the treatment of rheumatism by the salicylates, and he entered at once into the spirit of my inquiries and gave me every assistance in his power.

His statistics included seventy-seven cases of acute rheumatism treated by the remedy under consideration.

In forty-five of these cases, the action of the drug was so marked that its value could not be doubted.

In these 45 cases the average duration of the disease was only three days, the longest being seven, and the shortest one day.

But in the whole 77 cases there were only five in which the remedy proved an entire failure.

Three of these proved fatal, two from the accession of typhoid symptoms, and one from the supervention of a cerebral complication.

The two classes of cases least benefited by the drug, were those of moderate severity, and those in which there existed extreme hyperpyrexia.

In the last mentioned class of cases, the cold bath was added to the other treatment with excellent results.

Cardiac complications supervened in only two out of the 77 cases after the treatment was begun.

In the management of the patients, the plan pursued was to allow them to remain in the wards well covered up in bed for from one to three days, before any active treatment was instituted. This was done in order to observe whether the disease was one of a true rheumatic character, or only an abortive form of the complaint.

The doctor tested, in the treatment of these cases, salicin, salicylic acid and the salicylate of soda; and although each exercised a powerful influence in arresting the disease, he believes that the last mentioned preparation acted most energetically, and, as it is the most pleasant to the taste of the patient, as well as least disturbing to the stomach, he gives it the unqualified preference.

The plan pursued was to put the patient upon the salicylate of soda in thirty grain doses every four hours, until the temperature was reduced to the natural standard, and all other symptoms ameliorated.

After this result had been obtained, the remedy was given three times a day for a week or ten days, to prevent a relapse.

In looking over the records of the cases, I found the influence of the remedy upon the temperature of the patients was most marked. In 40 of them it was reduced to the natural standard within twenty-four hours, and never arose again.

If the remedy produced symptoms akin to cinchonism, the dose was reduced, but not discontinued.

In London I did not find any enthusiasm among those with whom I conversed on the subject, in favor of the salicylic treatment of rheumatism.

Even in so late a work on the Practice of Medicine as that of Dr. Bristowe, an article on acute rheumatism, only refers to the remedy in these words: " Salicylic acid in hourly doses of from

7 to 15 grains, has recently been largely employed, especially in Germany, with reputed success."

The author proceeds to recommend the use of free diluents in the form of lemonade, keeping his patient warm in bed, as a principal resource in the treatment of this disease.

When I arrived in Paris, I found the subject of the therapeutical effects of the salicylates under discussion before the Academy of Medicine, which discussion was elicited by a series of original articles read before that august body, by Prof. Germain Sée. These papers were all published in *La France Médicale*, and as I not only translated these, but also interviewed on several occasions, Prof. Guéneau de Mussy, of the Hôtel Dieu, I proceed to give below in a greatly condensed form, some of the facts elicited.

After a learned account of the discovery of salicin in 1830, by Leroux of Vitry, in France, the author of the paper dwells at some length on the different salicylates, as well as the diseases for which they have been given, and, summing up his experience in their use, says: "It is the salicylate of soda that I now almost exclusively employ."

A large number of experiments upon animals, to test the physiological effects of the remedy, are detailed at length.

These experiments demonstrate that it is a most powerful agent, and one that should be used with care.

Rabbits, in whose veins were injected from 1 to 2 grammes of the salicylate of soda, died after two or three of such injections, death being preceded by extreme dyspnœa and convulsions.

One case was reported of an old man, who died after administration of the drug in large doses, and in whose case, a suspicion at least arose, as to whether his death had not been produced by the medicine. The effects of the remedy on the temperature of animals were almost nil. In animals without fever, the remedy given in large doses, only lessened the temperature, on an average, 9-10°.

Furbringer injected pus into the blood of sixteen dogs, to produce septic fever, and then gave the salicylates to test their influence in lowering their temperature. It was slightly diminished in 9 cases out of 16.

Prof. Sée therefore concludes, that the effect of the remedy in abating the temperature of the body, is only such as is produced by its disturbing influence on the circulation and respiration.

Its action upon the kidneys is most marked, and it is probable that its efficacy in lowering the temperature in rheumatism, is due to its action in eliminating through these organs, the *materies morbi* upon which the disease depends.

Ten minutes after the medicine is given, it may be discovered in the urine by adding a very weak solution of the perchloride of iron to that fluid, giving a violet color.

It is also eliminated from the system so rapidly by the kidneys, that to keep up its effects, the doses must be frequently repeated.

So powerfully does it stimulate these organs that those who discussed the subject at the Academy were generally of opinion that it should be used with great care where any kidney lesion exists ; and, as a precautionary measure, we are recommended to test the urine before beginning the use of the remedy if there be the least suspicion of kidney disease.

Prof. Sée says on this subject: "If there exists any renal lesion it is necessary to prescribe the salicylic acid with great prudence, without which, even with a small dose, accidents may supervene."

The dose seems to be a matter of extreme importance; for, while the remedy is an active one, and may poison patients if given too largely, on the other hand, when given too sparingly, it may fail to produce any curative effect upon the rheumatism.

M. Sée gave his patients from eight to twelve grammes in the twenty-four hours. with the most happy results; while M. Oulmont, who gave only four grammes in the twenty-four hours, found no benefit from the remedy in the treatment of the same disease.

"If given in sufficiently large doses to produce its physiological effects (salicysm), we have inebriety, noises in the ears, and sometimes staggering."

We now pass to M. Sée's observations in "the treatment of rheumatism and gout by the salicylates."

After giving Stricker, of Germany, the credit of having first brought to the notice of the profession the use of these remedies in acute rheumatism, and acknowledging that his residence on the other side of the Rhine has prevented his views from being sooner adopted in France, he details his experience by an analysis of the following cases:

There were in all 52 patients suffering from acute rheumatism, 44 in the hospital and eight in private practice.

Six grammes of the salicylate of soda dissolved in 200 grammes of water were given five times in the twenty-four hours.

To quote M. Sée's exact words as to the results, he says: "Now in all these cases the duration of attacks treated by the salicylates did not exceed three days. There was not one single exception.

"The age of the patient did not change the result in the least.

"In the case of two children, the one 8 and the other 12 years of age, I prescribed the remedy in doses of from 1 to 3 grammes every hour, and the success was complete in two days.

"Rheumatism existing two, four, eight, and fifteen days was arrested at the end of two or three days.

"This is what we generally observe:

"The cessation of the pain; this generally abates in from twelve to eighteen hours, and the phenomenon was constant.

"The articular inflammation disappears in from one to three days.

"Movement again becomes free and easy by the third day.

"I have seen patients whose inferior extremities had been entirely immovable, get up at the end of two or three days."

On the necessity of continuing the treatment after the patient is apparently well, M. Sée says: "Nevertheless the treatment cannot be considered as complete, unless it be continued for ten or fifteen days at least; without which a relapse is inevitable. The reason of this is the rapidity with which the remedy is eliminated from the system.

"I sometimes suspended the treatment on purpose; and when the relapse occurred, I prescribed anew the same medicine, and the same effect followed as at first.

"I sometimes repeated this experiment two or three times on the same patient.

"I will conclude by saying that relapses never occur when we continue the treatment."

On the influence of salicylic treatment in the cardiac complications of rheumatism, M. Sée observes:

"Does the salicylic treatment exercise an influence favorable or unfavorable on the development or course of the complications and accompaniments so frequent in acute articular rheumatism?

"In those who entered the hospital in the three first days of the disease, I have not seen developed a single case of inflammation either of the pericardium or endocardium; and it may be logically inferred that, if the remedy 'strangles' the disease, we anticipate the invasion of the membranes of the heart."

He acknowledges, however, that the immunity from heart disease in the cases that he treated, is not corroborated by the German physicians who used the same remedy; but he thinks the reason for the difference is owing to the vacillating manner in which the latter employed the medicine at the outset.

In those patients who, from long continuance of the complaint before the treatment was begun, or from a previous attack of the disease, had already cardiac lesion, no influence seemed to be exerted by the salicylates as far as such complications were concerned.

M. Sée closes his remarks on the results of the salicylic treatment of acute rheumatism, by contrasting them with the records of 108 cases treated by the ordinary method now in vogue.

"Of these 108 cases, 10 had a duration of from 5 to 15 days; 58, from 16 to 20 days; 40, from 36 to 55 days, and upwards; therefore, 10 out of every 108 cases, get well in from 5 to 15 days, and 98 out of every 108, may expect their recovery on an average of 36 days."

Certainly if statistics are worth anything, these results are stupendous; in fact, nothing better can be desired.

In my interviews with M. Guéneau de Mussy, though I found him a warm advocate of the salicylates in rheumatism, his results had not been as flattering as those of M. Sée.

*Chronic Rheumatism.*—Though the number of cases recorded by M. Sée of this type of rheumatism treated by him, are not sufficiently large, perhaps, to warrant his enthusiastic deductions, yet, in view of the unsatisfactory character of the results that we generally obtain from any hitherto known remedies for the treatment of this disease, the results will certainly gain for his assertions a careful hearing and a disposition to test their accuracy.

The remedy was prescribed in the same dose as for the acute form of the disease. M. Guéneau de Mussy gives ten grammes three times a day of the salicylate of soda.

M. Sée says :

" My expectations were realized in a most happy manner. The attack disappeared exactly as in acute articular rheumatism, at the end of three or four days.

M. Bouchard has succeeded in four old men in the same happy manner.

" A patient who had had general chronic rheumatism for eleven years, confining him to his bed for from four to six months of each year, after six days' treatment was able to leave the hospital, all his joints unaffected.

" In five private patients, I observed exactly the same phenomena ; the tumefactions of the joints which had existed for several months, and in one for three years, disappeared at the end of six or eight days' treatment.

" The medicine was kept up for a month."

Of course M. Sée does not claim these brilliant results in patients whose joints have undergone extensive organic changes from the ravages of this disease ; but even in these, sub-acute paroxysms, to which they are so liable, can be promptly relieved by salicylic treatment.

*Gout.*—In the application of this remedy for the treatment of this disease, both in its acute and chronic forms, M. Sée's observations claim entire originality.

Not only do all the painful and inflammatory symptoms disappear, but also the chalky deposits about the joints are absorbed, and patients who have been such sufferers, that life had become a burden, walk forth after a few weeks use of the remedy, restored anew to health, and freed from one of the most painful disorders to which the human family is subjected.

*Lumbago and Sciatica.*—The fact elicited from the discussion of the therapeutic effects of this remedy at the Academy, were somewhat conflicting as to its efficacy in the above diseases; but the opinion prevailed that as the cause that produced them was often rheumatic in character, it should always be given an early trial in their management.

*Typhoid Fever.*—From the known properties of the drug in arresting septic changes when applied locally, as well as on account of its reputed value in lowering the temperature of the body, great hopes were entertained that in it we should find a valuable auxiliary to our other modes of treating this most formidable disease.

But most of those who took part in the discussions at the Academy of Medicine, were not enthusiastic as to the results that they had attained.

M. Sée took the ground that the medicine is not an antipyretic in the general acceptance of that term, and that the remedies given by the stomach, with a view to their acting antiseptically upon a portion of the intestines, so remote as that effected in this disease, could not be relied upon.

*Neuralgia.*—M. Sée found the drug act as a decided anodyne in most nervous diseases, and used it with success in a number of cases of the above disease.

He often substitutes it for the different preparations of opium with the happiest results.

W. S. Caldwell, M.D.,
Paris, October, 1877. *Of Warren, Ills.*

